# Clinical characteristics of redback spider bites

**DOI:** 10.1186/s40560-014-0062-3

**Published:** 2014-11-02

**Authors:** Toru Hifumi, Satoshi Fujimi, Takuya Yamagishi, Satoru Arai, Kyoko Sawabe, Akihiko Yamamoto, Manabu Ato, Keigo Shibayama, Akihiko Ginnaga, Nobuaki Kiriu, Hiroshi Kato, Yuichi Koido, Junichi Inoue, Masanobu Kishikawa, Yuko Abe, Kenya Kawakita, Masanobu Hagiike, Yasuhiro Kuroda

**Affiliations:** Emergency Medical Center, Kagawa University Hospital, 1750-1 Ikenobe, Miki, Kita Kagawa, 761-0793 Japan; Departments of Emergency and Critical Care, Osaka General Medical Center, 3-1-56 Bandaihigashi, Sumiyoshi-ku Osaka, 558-0056 Japan; Infectious Disease Surveillance Center, National Institute of Infectious Disease, Toyama 1-23-1, Shinjuku-ku Tokyo, 162-8640 Japan; Department of Medical Entomology, National Institute of Infectious Disease, Toyama 1-23-1, Shinjuku-ku Tokyo, 162-8640 Japan; Department of Bacteriology II, National Institute of Infectious Disease, Gakuen 4-7-1, Musashimurayama-shi Tokyo, 208-0011 Japan; Department of Immunology, National Institute of Infectious Disease, Toyama 1-23-1, Shinjuku-ku Tokyo, 162-8640 Japan; The Chemo-Sero-Therapeutic Research Institute (KAKETSUKEN), 1-6-1 Okubo, Kita-ku, Kumamoto-shi, Kumamoto 860-8568 Japan; Division of Critical Care Medicine and Trauma, National Hospital Organization Disaster Medical Center, 3256 Midori-cho, Tachikawa Tokyo, 190-0014 Japan; Division of Critical Care Medicine and Trauma, Yamanashi Prefectural Central Hospital, 1-1-1 Fujimicho, Kofu, Yamanashi 400-8506 Japan; Department of Emergency Medicine, Fukuoka City Hospital, 13-1 Yoshidukahonmachi, Hakata Fukuoka, 812-0046 Japan

**Keywords:** Redback spider, Antivenom, Systemic symptom

## Abstract

**Background:**

Redback spiders (*Latrodectus hasselti*) (RBSs) are venomous spiders that have recently spread to Asia from Australia. Since the first case report in 1997 (Osaka), RBS bites have been a clinical and administrative issue in Japan; however, the clinical characteristics and effective treatment of RBS bites, particularly outside Australia remains unclear. This study aimed to elucidate the clinical characteristics of RBS bites and to clarify the effectiveness of the administration of antivenom for treatment.

**Methods:**

We performed a retrospective questionnaire survey from January 2009 to December 2013 to determine the following: patient characteristics, effect of antivenom treatment, and outcomes. To clarify the characteristics of patients who develop systemic symptoms, we compared patients with localized symptoms and those with systemic symptoms. We also examined the efficacy and adverse effects in cases administered antivenom.

**Results:**

Over the 5-year study period, 28 patients were identified from 10 hospitals. Of these, 39.3% were male and the median age was 32 years. Bites most commonly occurred on the hand, followed by the forearm. Over 80% of patients developed local pain and erythema, and 35.7% (10 patients) developed systemic symptoms. Baseline characteristics, vital signs, laboratory data, treatment-related factors, and outcome were not significantly different between the localized and systemic symptoms groups. Six patients with systemic symptoms received antivenom, of whom four experienced symptom relief following antivenom administration. Premedication with an antihistamine or epinephrine to prevent the adverse effects of antivenom was administered in four patients, which resulted in no anaphylaxis. One out of two patients who did not receive premedication developed a mild allergic reaction after antivenom administration that subsided without treatment.

**Conclusions:**

Approximately one third of cases developed systemic symptoms, and antivenom was administered effectively and safely in severe cases. Further research is required to identify clinically applicable indications for antivenom use.

## Background

**Figure 1:**
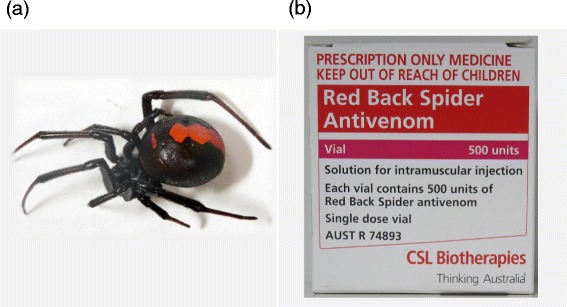
**Redback spider and an example of the antivenom. (a)** The left image shows a female Redback spider. **(b)** The right image depicts an example of the antivenom used for Redback spider bites.

Redback spiders (*Latrodectus hasselti*) (RBSs) are venomous spiders that produce the neurotoxin (alpha-latrotoxin) [1]. The adult female is characterized by a spherical black body with a prominent red stripe on the upper side of the abdomen (Figure 1a). Females have a body length of approximately 10 mm, and the male measures only 3–4 mm [2]. Although widely distributed in Australia, it has recently spread to Southeast and West Asia [3-5].

Symptoms of RBS bites are usually mild and localized, such as local pain and erythema. However, fatal cases had been reported before the development of antivenom (Figure 1b), which is manufactured by the immunization of horses [6,7]. Since the first case reported in Osaka in 1997, RBS bites have been a clinical and administrative issue in Japan [8,9]. Despite this, the clinical characteristics and optimal treatment of RBS bites, particularly outside Australia remain unknown.

Therefore, this study aimed to elucidate the clinical characteristics of RBS bites and the factors associated with developing systemic symptoms. We also aimed to clarify the effectiveness of the administration of antivenom for treatment.

## Methods

This is a retrospective observational study. The institutional review board of the Kagawa university hospital approved this cross-sectional, survey-based study (Heisei 26-029).

### Patients and setting

We prepared a questionnaire to examine the clinical characteristics of RBS bites in Japan. The questionnaires consisted of initial screening survey (phase I survey) and survey for clinical data (phase II survey). The initial screening questionnaire (phase I survey) was sent to 470 sentinel medical institutions originally used for the national surveillance for infections of antimicrobial resistant bacteria and severe influenza to cover major hospitals in all areas of Japan, such as University Hospitals, National Hospitals, and Red Cross Hospitals. The questionnaire was about the absence or presence of patients with RBS bites and was sent in January 2014 and collected by March 2014. Completed questionnaires were received from 297 (63.2%) sentinel medical institutions, with four hospitals that responded to having treated patients with RBS bites.

The questionnaire for obtaining clinical data (phase II survey) was sent to those four hospitals that responded to having treated patients with RBS bites in the phase I survey. We also sent the questionnaire (phase II survey) to seven other hospitals that possessed antivenom against RBS in May 2014. The surveillance period of the questionnaire spanned 5 years, i.e., from January 2009 to December 2013.

### Data collection

The following parameters were recorded: age, gender, date of injury, bite location, clinical symptoms (local pain, erythema, edema, sweating, headache, nausea, abdominal numbness, systemic pain, and others), vital signs (systolic blood pressure and body temperature), laboratory data (white blood cell and platelet counts, creatinine kinase, and aspartate aminotransferase), treatment-related factors (analgesics and antivenom), effectiveness and adverse effects of antivenom, and outcomes (days in hospital, days in intensive care unit (ICU), and in-hospital mortality).

### Diagnosis of RBS bites and definition of systemic symptoms

No definite diagnostic criteria exist. Diagnosis of RBS bites was based on either the patient’s history or the positive identification of RBS presented by the patient. Systemic effects were considered to include sweating, headache, nausea, abdominal numbness, systemic pain, fever, hypertension, parasthesia, fasciculations, and cardiac effects [10]. In the current study, patients with systemic symptoms were defined as those who developed at least the abovementioned one symptom.

### Treatment of RBS bites

The definitive treatment for RBS envenomation in Australia is the use of a specific RBS antivenom produced by Commonwealth Serum Laboratories (CSL) [11]. Because RBS antivenom has not been approved by the Ministry of Health, Labour and Welfare in Japan, clinicians have to privately purchase and import it from CSL. In Australia, the indications for RBS antivenom are patients with signs of systemic envenomation, those with pain not controlled with simple analgesia, or for those who require repeated doses of opiates [12]. In the current survey, the decision to administer antivenom was made by individual doctors and was not based on any protocol.

### Primary data analysis

Patient characteristics, treatment-related factors, and outcomes were compared between the localized and the systemic symptoms groups using Mann-Whitney *U* test and the Fisher’s exact test for categorical variables, as appropriate.

Two-tailed *P*-values of ≤0.05 were considered statistically significant. Statistical analysis was performed using JMP version 11 (SAS, Cary, NC, USA).

## Results

### Demographic data and clinical characteristics of all study patients

**Figure 2:**
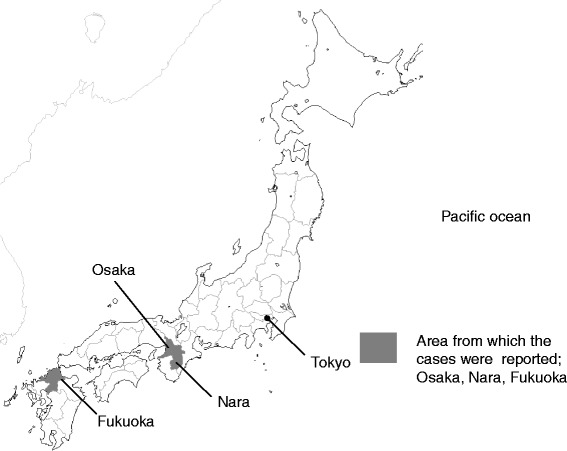
**A map showing the relative locations of cases of redback spider bites: Osaka, Nara, and Fukuoka Prefectures.**

Over the 5-year study period, 28 patients were identified from 10 hospitals. The areas where RBS bites were reported were limited to three prefectures: Osaka, Nara, and Fukuoka (Figure 2). The patient characteristics are summarized in Table 1; 39.3% were male and the median age was 32 years. The most common sites for bites were the hand (42.9%) and the forearm (17.9%). Over 80% of patients developed local pain and erythema, and systemic symptoms occurred in 10 patients (35.7%). Antivenom was administered to six patients, four (14.3%) were admitted to hospital, and one required care at ICU. All patients recovered without lasting adverse effects.

**Table 1:** **Population characteristics (** ***n*** **= 28)**

**Characteristics**	**Values**
Age (years)	32 (15.5–56.5)
>65 years	4 (14.3%)
<15 years	6 (21.4%)
Gender, male, n (%)	11 (39.3)
Bite site	
Hand	12 (42.9)
Forearm	5 (17.9)
Clinical symptoms	
Local	
Local pain, *n* (%)	25 (89.2)
Edema, *n* (%)	13 (46.4)
Erythema, *n* (%)	24 (85.7)
Systemic symptoms	10 (35.7)
Sweating, *n* (%)	2 (7.1)
Headache, *n* (%)	2 (7.1)
Nausea, *n* (%)	4 (14.8)
Numbness on the abdomen, *n* (%)	2 (7.1)
Systemic pain, *n* (%)	2 (7.1)
Others (high grade fever at 39°C light headedness), *n* (%)	2 (7.1)
Vital signs on admission	
SBP (mmHg)	132 (123–150)
BT (°C)	36.8 (36.4–37.1)
Laboratory data	
WBC (/mm^3^)	9,000 (6,597–9,600)
Platelet count (×10^4^/mm^3^)	23.6 (17.8–28.9)
CK (IU/L)	156 (73–170)
AST (IU/L)	33 (22–52)
Treatment	
Antivenom, *n* (%)	6 (21.4)
Analgesics, *n* (%)	8 (28.6)
Outcome	
Hospital admission, *n* (%)	4 (14.3)
ICU admission, *n* (%)	1 (3.6)
Mortality, n (%)	0 (0)

Data are presented as median (interquartile range, IQR) for continuous variables and *n* (percentage) for categorical variables.

*SBP* systolic blood pressure; *BT* body temperature; *WBC* white blood cell; *CK* creatine kinase; *AST* aspartate aminotransferase.

### Comparison between the localized and systemic symptoms groups

We compared the clinical characteristics between the localized and systemic symptoms groups to clarify the characteristics of patients that develop systemic symptoms; our results are summarized in Table 2. There were no significant differences between the two groups in terms of baseline characteristics, vital signs, laboratory data, treatment-related factors, and outcomes.

**Table 2:** **Comparison between the groups with local and systemic symptoms**

	**Limited to local (** ***n*** **= 18)**	**Systemic (** ***n*** **= 10)**	***P*** **value**
Age (years)	30.5 (10.8–52.3)	32.5 (18.5–59.8)	0.49
>65, *n* (%)	3 (16.7)	1 (10)	1.00
<15, *n* (%)	5 (27.8)	1 (10)	0.37
Gender, male, *n* (%)	6 (33.3)	5 (50)	0.44
Bite site			0.12
Hand	11 (61.1)	1 (10)	
Forearm	2 (11.1)	3 (30)	
Other/unknown	5 (27.8)	6 (60)	
Vital signs on admission			
SBP (mmHg)	135 (111–156)	130 (125–136)	0.67
BT (°C)	36.9 (36.4–37.1)	36.7 (36.5–36.8)	0.59
Laboratory data			
WBC (/mm^3^)	7,697 (5,398–9,550)	9,000 (7,350–9,750)	0.46
Platelet count (×10^4^/mm^3^)	23.3 (17.8–30.4)	23.6 (18.0–28.9)	0.88
CK (IU/L)	123 (65–169)	159 (85–190)	0.62
AST (IU/L)	35 (23–51)	28 (22–61)	0.77
Treatment			
Antivenom, *n* (%)	2 (11.1)	4 (40)	0.15
Analgesics, *n* (%)	5 (27.8)	3 (30)	1.00
Outcome			
Hospital admission, *n* (%)	1 (5.6)	3 (30)	0.12
ICU admission, *n* (%)	0 (0)	1 (10)	0.36

Data are presented as median (interquartile range, IQR) for continuous variables and *n* (percentage) for categorical variables.

*SBP* systolic blood pressure; *BT* body temperature; *WBC* white blood cell; *CK* creatine kinase; *AST* aspartate aminotransferase.

### Details of cases who received antivenom

The details of six patients who received antivenom are summarized in Table 3. Antivenom administration relieved symptoms in four patients who developed systemic symptoms. Premedication with an antihistamine or epinephrine to prevent the adverse effects of antivenom was administered in four patients, which resulted in no anaphylaxis. One out of two patients who did not receive premedication developed a mild allergic reaction after antivenom administration that subsided without treatment.

**Table 3:** **Cases administered with antivenom**

**Case**	**Age**	**Gender**	**Symptoms**	**Reason for administration**	**Premedication**	**Adverse effect**	**Clinical effect**
1	6	M	Localized	N/A	Antihistamine	None	N/A
2	14	M	Systemic	Systemic symptoms (numbness on the abdomen)	Antihistamine	None	Pain relief
3	36	M	Systemic	Systemic symptoms (headache)	None	Flushing on the face	Pain relief
4	59	F	Systemic	Systemic symptoms (systemic pain, dizziness, nausea)	Epinephrine	None	Symptoms relief
5	68	F	Localized	Patient’s wish	Antihistamine	None	N/A
6	87	F	Systemic	Systemic symptoms (severe systemic pain)	None	None	Pain relief

*N/A* not applicable.

## Discussion

In the current survey, all 28 cases recovered well. Six cases received antivenom, of which four had symptomatic relief with no serious adverse effects. One out of two patients who did not receive premedication developed a mild allergic reaction after antivenom administration that subsided without treatment. Notably, 36% of patients developed systemic symptoms. No significant factors associated with systemic symptoms were identified.

In Australia, antivenom is recommended for patients with signs of systemic envenomation. Indeed, those with severe or systemic symptoms and patients at greater risk, such as children, pregnant women, and the elderly, are more likely to receive antivenom [12,13]. Conversely, no indication has been provided for antivenom use in clinical practice in Japan. Five out of six cases in the current survey (four cases with systemic symptoms and one pediatric case) received antivenom based on the indications used in Australia. Although four cases out of ten that developed systemic symptoms recovered with RBS antivenom, the remaining cases with systemic symptoms recovered without antivenom. We identified no cases among pregnant women. Given these facts, further research is required to identify the appropriate clinical indications for antivenom use in Japan.

Alpha-latrotoxin causes synaptic vesicle exocytosis from the presynaptic terminal, via a calcium-dependent mechanism, leading to the release of catecholamines and acetylcholine [14]. Therefore, although the primary impact of the envenomation can be mild, it is assumed that these substances, together with hypertension induced by persistent pain, worsen the condition among both elderly patients with comorbidities and pregnant women. In such populations, antivenom administration may be considered.

RBS antivenom is manufactured by the immunization of horses. Therefore, there is a risk of adverse events such as anaphylaxis and serum sickness disease [15,16]. In studies in Australia, allergic reactions to the antivenom have been rare (<2%) [7]. However, Mamushi antivenom, which is also manufactured by the immunization of horses, causes a 2.4%–9% rate of anaphylactic reactions in Japan [17,18]. In the present study, none of the four cases that received antivenom with premedication against anaphylaxis had an adverse reaction. However, one case that did not receive premedication developed a mild allergic reaction. Therefore, premedication with an antihistamine and/or epinephrine should be used when the perceived benefit is greater than the risk of adverse effects.

The serious concern with the current treatment of RBS bites is that RBS antivenom is not approved by the Ministry of Health, Labour and Welfare. Therefore, clinicians are required to privately import it from Australia. Moreover, in 2013, all imports from Australia were suspended due to the low production of RBS antivenom by CSL. In 2013, the Ministry of Health, Labour and Welfare of Japan launched a research group to evaluate the safety and efficacy of antivenom and to organize and maintain information on RBS bites [19]. In the group, domestic production of RBC antivenom was carefully discussed, and this production started since April 2014.

There are many limitations to this study. A major limitation is that it had a retrospective design and a relatively small sample size. Selection bias may also have occurred because not all cases were collected. We conducted the current survey with 470 sentinel medical institutions originally used for the national surveillance for infections of antimicrobial resistant bacteria and severe influenza with response rate of 63.2%. Many cases may have remained undiagnosed or misdiagnosed because of the unfamiliar symptoms presented by RBS bites. Given the number of patients included, multivariate analysis (logistic regression model) could not performed to identify the factors associated with developing systemic symptoms.

## Conclusions

Approximately one third of cases developed systemic symptoms and antivenom was administered effectively and safely in severe cases. Further research is required to identify clinically applicable indications for antivenom use.
